# Challenges in Healing Wound: Role of Complementary and Alternative Medicine

**DOI:** 10.3389/fnut.2021.791899

**Published:** 2022-01-20

**Authors:** Prakash Monika, Mathikere Naganna Chandraprabha, Annapoorni Rangarajan, P. Veena Waiker, Kotamballi N. Chidambara Murthy

**Affiliations:** ^1^Department of Biotechnology, M.S. Ramaiah Institute of Technology, Bangalore, India; ^2^Department of Molecular Reproduction, Development and Genetics, Indian Institute of Science, Bangalore, India; ^3^Department of Plastic Surgery, Ramaiah Medical College and Hospitals, Bangalore, India; ^4^Central Research Laboratory and Division of Research and Patents, Ramaiah Medical College and Hospitals, Bangalore, India

**Keywords:** acute wound, chronic wound, complementary and alternative medicine, infection, phytochemicals, polyphenols

## Abstract

Although the word wound sounds like a simple injury to tissue, individual's health status and other inherent factors may make it very complicated. Hence, wound healing has gained major attention in the healthcare. The biology wound healing is precise and highly programmed, through phases of hemostasis, inflammation, proliferation and remodeling. Current options for wound healing which includes, use of anti-microbial agents, healing promoters along with application of herbal and natural products. However, there is no efficient evidence-based therapy available for specific chronic wounds that can result in definitive clinical outcomes. Under co-morbid conditions, chronic would poses numerous challenges. Use of Complementary and Alternative Medicines (CAMs) in health care sector is increasing and its applications in wound management remains like to “*separate the diamonds from ore*.” Attempts have been made to understand the wound at the molecular level, mainly through the analysis of signature genes and the influence of several synthetic and natural molecules on these. We have outlined a review of challenges in chronic wound healing and the role of CAMs in chronic wound management. The main focus is on the applications and limitations of currently available treatment options for a non-healing wound and the best possible alternates to consider. This information generates broader knowledge on challenges in chronic wound healing, which can be further addressed using multidisciplinary approach and combination therapies.

## Graphical Abstract

Challenges in healing wound: role of complementary and alternative medicine. 
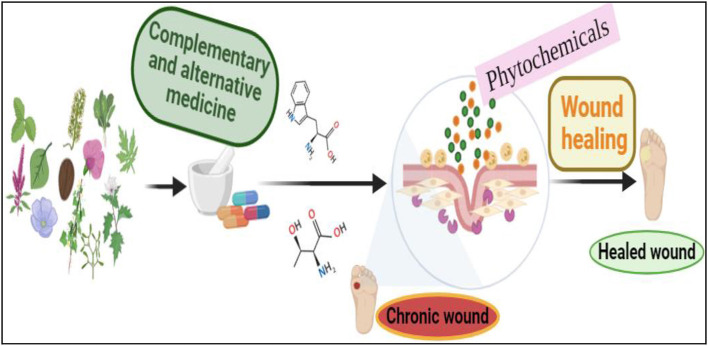

## Introduction

Use of natural medicine and principles for healing of one of the fundamental principles of traditional medicine. Although majority of them are not in mainstream due to lack of evidences, some of them have successfully entered clinical utility after gaining significance in different stages of research for inflammation, cancer and other chronic diseases. This is mainly due to unique ability of natural molecules to interact with different bio-molecules. Therefore, both understanding the critical cellular components altered during wound and progressions of wound as well as molecules to reverse/repair those changes are vital. In this direction, there is a huge potential for development of safe, sustainable and effective treatment module using alternate medicine from plant or other natural sources. This will help to scientifically promote several traditional medicines for healing different category of wounds.

The incidence and burden of wound is one of the major health care concerns, which accounts for more than more than 8.2 million people were suffered from wound in US and cost of care ranged from 28.1 to 96.8 billion$. The global wound care market size was valued at USD 18.4 billion in 2018 and is expected to grow at a compound annual growth rate (CAGR) of 3.9% from 2019 to 2026 (1). Chronic wounds are greatest burden to health care and account for expenditure of more than 25 billion US$ per year and affect over 6.5 million people in US alone (2). These cause burden through, prolonged hospitalization, loss of mobility, compromising quality of life, requirement of support and sometime increase the risk of nosocomial infection causing burden to health care workers and other patients visiting hospital (3). The recent pandemic is adding additional burden due to health care which is mainly due to limited access to healthcare facilities. Clinical challenges in wound treatment are many, clear understanding on molecular signatures is expected to provide better option for clinicians to select drug or treatment regime.

Literature on wound biology, healing and role of CAM in wound healing was searched in all science databases such as PubMed, Web of Sciences, Science direct and Google scholar. Information with authenticated references and sufficient data as evidence were considered for literature search. The period of literature collection was for last 45 years for treatment modules and others relevant to recent developments.

## Wound and Wound Microbiology

A wound is defined as the breakage in the continuity of the skin. The structure of the skin is complex and wound biology is understood by knowing the factors influencing the local physiological environment. Many local conditions influence wound occurrence, persistence, and healing. The most common condition that is representative of a wound is microbial infection and inflammation. Exposed skin surface which comprises of subcutaneous tissue helps wide range of microorganisms to colonize on the substratum. The microflora of chronic wounds is far more complicated than previously appreciated, as they have complex structures and wide range populations (4). Both aerobic and anaerobic microorganisms are present in acute and chronic wounds. However, anaerobic microorganisms are often undiscovered due to various reasons as detailed elsewhere (5), and is not the focus of this article. Further, the prevalence of contamination by microorganisms also depends on a person's immunity or body-host defense. If the host immune response is compromised, involved tissue is devitalized by ischemic, hypoxic, or necrotic conditions that favor microbial growth (5).

Duerden (6) described in his study that the microbial wound contaminants can originate from three main sources: (i) the environment (exogenous microorganisms in the air or those introduced by traumatic injury), (ii) the surrounding skin (involving members of the normal skin microflora such as *Staphylococcus epidermidis*, micrococci, skin diphtheroid, and Propionibacterium), and (iii) endogenous sources involving mucous membranes (primarily the gastrointestinal, oropharyngeal, and genitourinary mucosae).

## Wound Healing and Wound Molecular Biology

Wound healing is a complex dynamic process involving several sequential steps, including induction of inflammatory process, regeneration of parenchyma tissue, migration and proliferation of parenchymal tissue cells, production of extracellular matrix proteins, remodeling of tissue and gaining wound strength (7). Healing of a wound involves different cell types, secreted growth factors, cytokines, the extracellular matrix and various enzymes. Various cells such as platelets, neutrophils, monocytes, macrophages, fibroblasts, keratinocytes, endothelial cells, epithelial cell and myofibroblasts are involved in wound healing process. Among all cells, fibroblasts have long been recognized as key cells in wound healing as they play a major role in all the three phases (3). There are various factors that affect the wound healing process such as microbial infection or biofilm formation. In addition to this, ischemia and reperfusion play an influential role in wounded skin. Thomas et al. (8) explained the molecular biology involved in ischemia and reperfusion that are major causes for the pathological condition in a wound. Ischemia-reperfusion injury is characterized by a sequence of biochemical and cellular events that causes cell damage extensively through pathways that lead to leukocyte and complement activation, oxidative stress, and microvasculature dysfunction.

Besides, many molecular factors that include growth factors and receptors (platelet-derived growth factor (PDGF), transforming growth factor-beta (TGF-ß); cytokines (fibroblast growth factor (FGF), tumor necrosis alpha (TNFα) and interleukin-1 (IL-1); enzymes (matrix metalloproteinases (MMPs), tissue inhibitors of MMPs, seperinase) often have multiple and overlapping functions in normal wound healing (9). In addition, Angiogenesis plays a critical role in wound healing. By developing capillary sprouts, that digest endothelial cells they invade the extracellular matrix (ECM) stroma after penetrating through the underlying vascular basement membrane, that later forms tube-like structures that continue to extend, branch, and form networks (10). Various angiogenic stimulators such as vascular endothelial growth factor (VEGF), TGF-ß, TNFα, PDGF, FGF, angiogenin and angiopoietin-1 are known to stimulate wound healing in various stages such as angiogenesis initiation, angiogenesis amplification, vascular proliferation, vascular stabilization and angiogenesis stabilization (10). An occurrence of wound follows the activation of these molecular factors, whereas a wound is persisted if the complex set of interactions between these molecules is devastated. Understanding the causes of altered expression of such molecular factors that lead to modified cellular function, will provide an insight into potential points for intervention.

## Wound Healing in Acute and Chronic Conditions

Wounds can be classified into two broad types, acute wounds, and chronic wounds. Acute wounds heal normally in a very orderly and efficient manner. They are characterized by four distinct, but overlapping phases: haemostasis, inflammation, proliferation and remodeling (11). These wounds progress through the normal stages of wound healing such as inflammation, proliferation and remodeling and show definite signs of healing within 2–4 weeks. In contrast, chronic wounds do not follow the sequential stages of healing (they often get “stalled” in one phase) and fail to show evidence of healing within 4 weeks (12).

During normal physiological processes such as wound healing (as in case of acute wounds), inflammatory cells are recruited to the site of injury and help in tissue repair through secretion of cytokines and growth factors that promote tissue remodeling and angiogenesis. Angiogenesis is impaired in all chronic wounds leading to further tissue damage resulting in chronic hypoxia and impaired micronutrient delivery. Vasculopathies associated with diabetes include abnormal blood vessel formation (e.g., retinopathy, nephropathy), decreased angiogenesis and accelerated atherosclerosis leading to coronary artery disease, peripheral vascular disease, and cerebrovascular disease (13). Of all the angiogenic stimulators, VEGF plays a crucial role in wound healing. Many studies have shown that diabetic chronic wounds have deficient VEGF and application of VEGF stimulates healing of chronic wounds in animal models (14, 15). On the other hand, chronic venous stasis ulcer patients have an elevated levels of VEGF in their circulation (16). Since many factors regulate wound angiogenesis, it is important to understand various cellular and molecular events in angiogenesis that are dysregulated in non-healing chronic wounds.

In normal wound healing, inflammation subsides once the tissue repair is completed. In contrast, the regulation of inflammatory cells and cytokines are circumvented during neoplastic progression which has resulted in the characterization of tumors as wounds that never heal (17, 18). Chronic inflammation, a hallmark of the non-healing wound, may ultimately predispose these wound sites to potential malignant change (19). Thus, in the case of chronic wounds, it is obvious that attempting to understand and determine the underlying cause of failure to progress in a timely fashion through the wound healing stages is the key to turn a chronic wound into a healing wound, and also divert it from neoplastic progression.

## Biological Factors in Non-Healing Chronic Wounds

Many factors can impair the healing process. Specific biological markers characterize the non-healing of chronic wounds. Both local and systemic factors contribute to delayed healing. Local factors include the presence of tissue maceration, foreign bodies, biofilm, hypoxia, ischemia, and wound infection. Systemic factors include diabetes, advanced age, malnutrition, and other chronic organ diseases. However, it is impossible to completely remove or reduce the impact of these factors even by good clinical practice. In addition to local and systemic factors that impair healing, reduced tissue growth factors, increased proteolytic enzymes such as matrix metalloproteinases that degrade extracellular matrix, increased inflammatory mediators such as over-abundant neutrophil infiltration (as in case of pressure ulcers), and the presence of senescent cells could be potential biomarkers for chronic wounds (11).

Non-healing chronic wounds are also characterized by myofibroblasts activity that persists and drives tissue alterations, which is particularly evident in hypertrophic scars developing after burn injury and in the fibrotic phase of scleroderma (3, 20). Myofibroblasts-generated contractions are also typical for fibrosis, affecting vital organs such as the liver, heart, lung and kidney (21). The hypertrophic scar is characterized by hypervascularization, aberrant deposition of ECM molecules and overabundant collagen accumulation (22). It is the chronic wound such as long-time exposure to toxic chemicals including carcinogens that produces damaging effect on the functionality of chemo-surveillance. Wound healing may proceed in a relatively unimpeded manner for many patients with cancer, due to malnutrition, nature and effects of the oncologic disease process and its treatment methods (23). A recent study by Liau et al. (24) reported that cancer due to non-healing chronic wound is common phenomenon. Thus, the best strategy to win the war on cancer is to restore the functionality of chemo-surveillance, increase nutritive food uptake and prevent the loss of wound healing metabolites that ultimately results in enhanced wound healing (24). Dysregulations in cell-intrinsic and extrinsic metabolism also affect the wound healing process leading to non-healing chronic wounds (25). Understanding deregulated and defective cellular mechanism may allow the development of therapeutic approaches to activate latent regenerative capacities and enforce a cadre of endogenous repair mechanisms. Thus, knowledge of various biological factors involved in chronic wounds is essential to develop specific therapeutic drugs. However, more research needs to be carried out that substantially supports the translatability of findings in experimental models to human wound scenarios.

## Clinical Presentation and Challenges in Chronic Wounds

Chronic wounds represent a major health care burden including financial expenses and have a devastating impact on morbidity. In India, public healthcare funding has been reported at 5% of the annual gross domestic product, with more than 80% of healthcare costs met from out-of-pocket payments (26). These do not reflect the economic loss, frustration, and impaired quality of life experienced by chronic wound patients. Thus, they remain a major clinical challenge in long-term care impacting the quality of life for patients and health care costs (27, 28).

**Figure 1:**
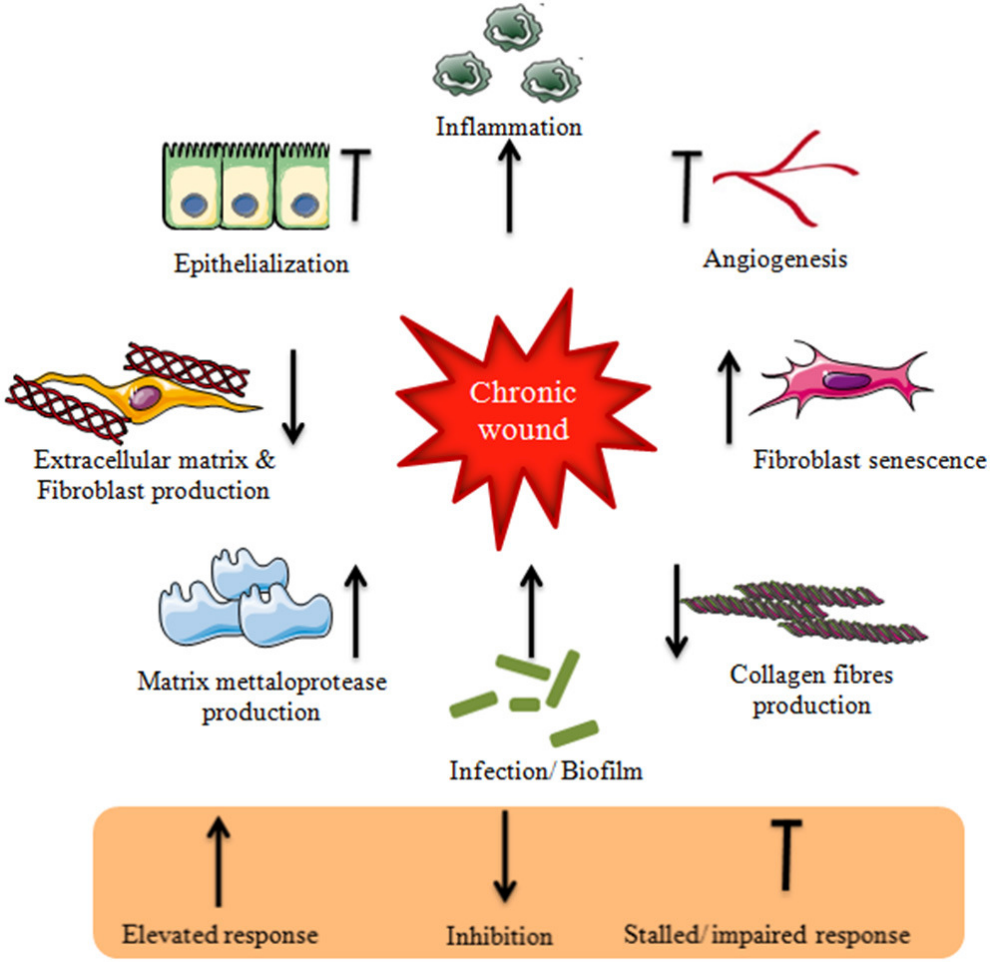
Schematic representation of challenges in chronic wound environment.

Chronic wounds are often characterized by pathologic responses resulting in fibrosis and non-healing chronic ulcers. This clinical condition is a result of undiagnosed or untreated wounds. The major clinical signs and symptoms of a patient with a chronic wound are pain, erythema, edema, heat, purulence with high wound bioburden (29). Furthermore, signs and symptoms specific to secondary wounds often observed in proliferative phase include: (1) wound breakdown (2) serous drainage with concurrent inflammation, (3) foul odor (4) pocketing at the base of the wound (5) discoloration of granulation tissue, (6) friable granulation tissue, (7) delayed healing (30). However, these signs and symptoms need to further be validated for effective chronic wound management and treatment. A study conducted by Sue et al. (30)to validate the clinical symptoms of the chronic wound showed signs specific to secondary wounds were better biomarkers of chronic wound infection than the classical signs with a mean sensitivity of 0.62 and 0.38, respectively. Besides, increasing pain and wound breakdown were both sufficient indicators with the specificity of 100%. These signs and symptoms can in turn be correlated to conditions that represent its physiological environment. Thus, it is understood that chronic wounds face a challenging environment that triggers the wound to a non-healing condition (Figure 1). It is utmost necessary to understand in depth the underlying mechanisms involved in its pathologic conditions. This is possible by identifying the clinically relevant biological markers that are key factors for the chronicity of a wound. Thus, by identifying a biomarker and targeting the same (as a prognostic, diagnostic, or treatment option) would potentially help in reversing the status of chronic wounds (Table 1).

**Table 1:** Clinically relevant biological markers to be targeted to reverse the status of chronic wounds.

**Biomarker**	**Characteristics in chronic wound**	**Method of analysis**	**Possible solutions**
Protein levels	A decrease in total protein content	Bradford protein assay	Increase dietary protein intake
Proteinase levels	Elevated metalloproteinases levels, especially MMP 2, 7 and 9	Sodium dodecyl sulfate-polyacrylamide gel electrophoresis (SDS PAGE), Human MMP diagnosis kit	Increase the levels of tissue inhibitors of MMPs
Tissue bacterial levels	High tissue levels of a diverse range of bacteria. Bacteria release enzymes that reduce growth factors and produce MMPs that degrade the extracellular matrix. Increased inflammatory response is observed	Microbiological tests	Levels of bacterial counts can be decreased by continuous removal of the exudates, thus reducing inflammation
Genes	Significant differences in the expression of a diverse collection of genes (can be up regulated or down regulated)	Real-Time quantitative reverse Transcription Polymerase Chain Reaction (qRT PCR), Microarray	Target specific genes for regulation using natural or synthetic products
Growth factors	Reduction in growth factor levels necessary for healing	Enzyme-linked immunosorbent assay (ELISA)	Increase fibroblast production and proliferation which in turn release necessary growth factors
Cytokine levels	Increased proinflammatory cytokine levels	Column chromatography	Vitamin D treatment and elemental diet can reduce the proinflammatory cytokine levels
pH	Rate of wound healing was found to be lower at elevated alkaline pH as compared to wounds having pH close to neutral	Glass top electrode	Lowering the pH to more acidic environment using pH modulating topical agents
Hypoxia	Partial Oxygen Pressure of <30 mmHg. It impedes fibroblast proliferation and collagen production affecting wound healing by allowing certain negative entities, such as bacteria, to flourish.	Pulse oximeter, laser doppler flow, skin perfusion pressure and ankle brachial index	Vacuum-assisted closure (negative pressure wound therapy), Compression bandages or compression garments
Poor nutrition	Wounds are characterized by prolonged inflammatory phase of healing. Decreased fibroblast formation is observed as long as the patient remains in a catabolic state due to poor nutrition.	Nutrition analysis and analysis of daily food intake	Improving patient's diet by providing nutritious food intake or dietary supplements

Chronic wound often characterized by infection/ biofilm elevates the production of inflammatory and proinflammatory cytokines that result in an increased production of matrix metalloproteases and a reduction in their inhibitors. The prolonged inflammation results in destruction of extracellular matrix and fibroblast production. Due to inhibition of fibroblast production, the collagen fibers necessary for wound remodeling are scarcely produced. The chronic wound associated fibroblasts show fibroblast senescence. Chronic wound environment impairs angiogenesis and stalls epithelialization. These characteristics represent chronic wound environment that cause delayed healing.

It has been estimated that ~1% of the population will develop leg ulceration in the course of their lifetime (31, 32). Indian studies on the epidemiology of chronic wound estimated the prevalence at the rate of 4.5/1,000 population (33). Untreated or inadequately treated acute traumatic wounds are a frequent cause of these chronic wounds. Wound associated with chronic diseases such as diabetes and cancer pose a greater challenge to treat and manage, which is mainly due to molecular complexity.

It is understood that diabetes affects wound healing through the decreased inflammatory responses, loss of protective sensations due to neuropathy, the development of ischemia, and increased risk of infection (34, 35). Besides, chronic non-healing pressure ulcers are serious and exhibit frequent occurrence (as in the case of spinal cord injury) among the immobile and debilitated patients (11). Based on this information, to identify the factors responsible for delayed wound repair, it is essential to understand wound healing activity at the molecular level. Thus, current research for treating chronic wounds focuses on understanding the underlying molecular mechanisms in clinically different wounds and at various stages of their progression and healing.

## Complications During Treatment of Chronic Wounds

Most of the chronic wounds are characterized by bacterial contamination and further biofilm formation which creates complications during the treatment. In addition to elevated inflammatory cells and the production of pro-inflammatory cytokines (as discussed earlier), a bacterial infection is very common. Bacterial infection in wounds depends on the organisms present, their virulence rate, and their resistance to host. Hence, it is utmost critical to detect which organism is responsible for the infection to treat with a specific antibiotic. However, the use of antiseptics, topical, and oral antibiotics can increase the risk of future antimicrobial resistance such as Methicillin-resistant Staphylococcus aureus (MRSA) and requires further assessment (36). Systemic infections are usually treated using systemic antibiotics. However, once there is a control in bacterial balance, the use of topical antibiotics should be discontinued, as prolonged courses of antibiotics may hamper wound healing and lead to antimicrobial resistance (37).

Another study assessed wound pH of the wound bed to track the wound healing progress. Though there was some evidence to differentiate between a healing and a non-healing wound using pH as a biological tracker, treatment options using knowledge on wound environmental pH need to still be explored (38).

**Figure 2:**
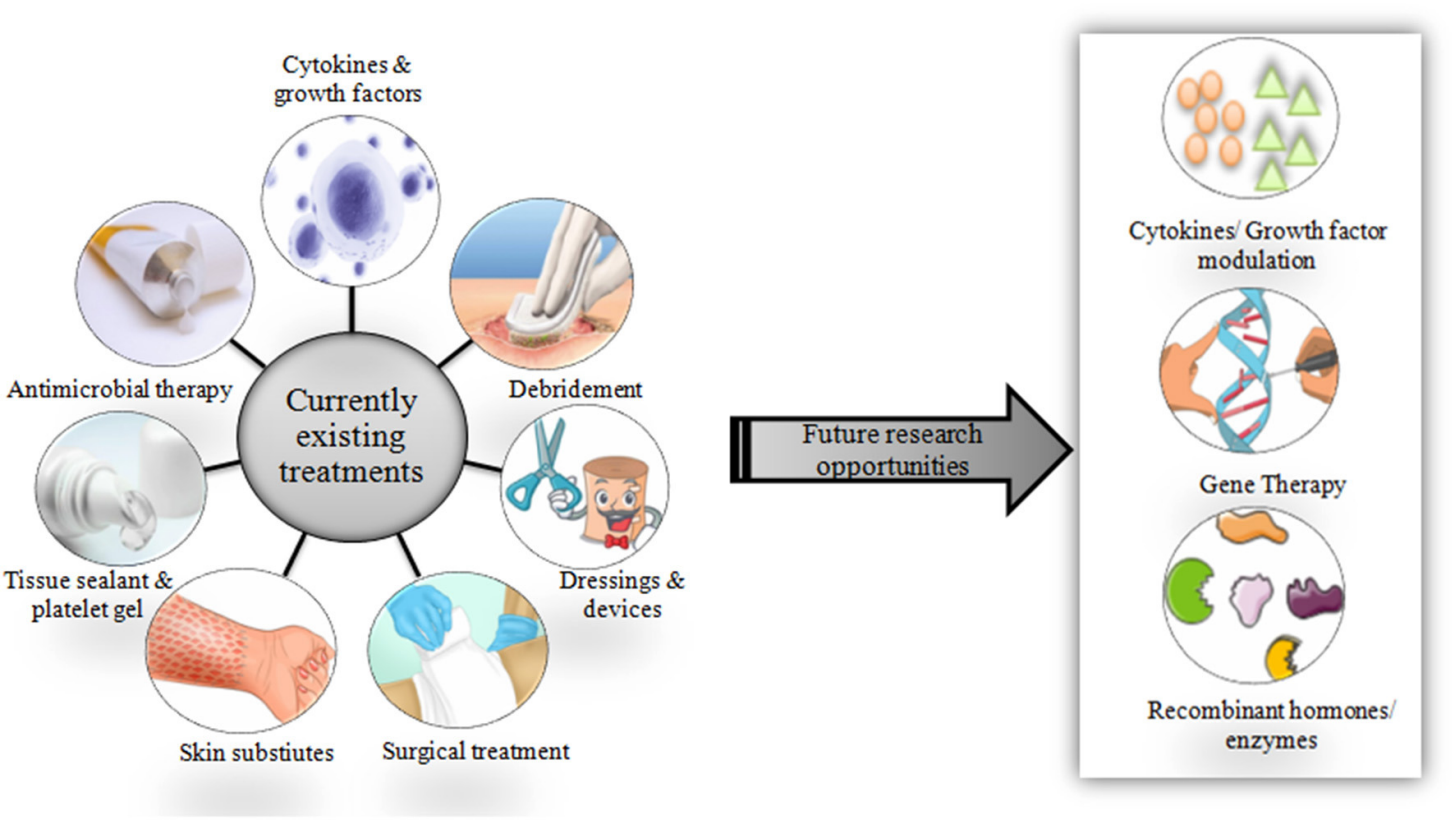
Schematic representation of currently available therapies and scope for future treatment options.

Studies (*in vitro*, preclinical, and clinical) on chronic wound diagnosis and treatment have substantially increased in the past decade. However, very shreds of evidence show the conductance of quality randomized controlled trials. Laser therapy and phototherapy fail to statistically improve chronic ulcer healing (39). Additionally, the development of wound diagnostic instruments is still in its infancy. A sophisticated analytical instrument such as Magnetic resonance imaging is not suitable for routine clinical purposes. There are wide varieties of treatment options available and are currently in use (Figure 2).

Novel approaches using emerging technologies could help in wound care. Cytokines and growth factors that play a very important role in wound healing can be modulated as required in the case of chronic wounds. By doing this, the imbalanced production of cytokines and growth factors can be controlled to normal. Gene therapy may allow genes or gene-derived messengers that possess wound healing properties to be delivered specifically at the wound site at directed time points, desired dose, and during a specific phase of healing (40–42). Skin and composite equivalents derived from embryonic stem cells in addition to application of bone marrow-derived stem cells may serve as possible options in near future (43).

Future developments could also target recombinant enzymes and hormones that help in wound repair. However, besides their uses and advantages, currently available treatment methods (including both medical and surgical) face a lot of limitations such as short residence time, low efficacy, high toxicity, high costs and high risk of infection as discussed in Table 2. This is because chronic wounds remain unresponsive to conventional wound care treatments such as topical agents, wound dressings, and skin grafts. These drawbacks have necessitated the need to focus and explore more on the role of CAMs in the treatment of non-healing chronic wounds, which could potentially provide reliable solutions in the near future.

**Table 2:** Comprehensive details on different kinds of treatment options currently in use for wounds with their uses and limitations.

**Category of dressing**	**Available treatment**	**Uses in medical & surgical purposes**	**Limitations**	**Reference**
Traditional dressings	Topical liquid formulations (povidone iodine)	Used in initial stages of wound healing for reducing bacterial load; Debriding and de-sloughing agents	Short residence times on the wound site, especially where there is a measurable degree of suppuration (exuding) of wound fluid	(44, 45)
	Topical liquid formulations (saline solutions)	Wound cleansing agent; Irrigate dry wounds		(45)
	Semi-solid preparations (silver sulphadiazine cream and silver nitrate ointment)	Treat bacterial infection	Not very effective at remaining on the wound area as they rapidly absorb fluid, lose their rheological characteristics and become mobile.	(46, 47)
	Solid materials (cotton wool, natural or synthetic bandages and gauzes)	Used as primary or secondary dressings	They are dry and cannot provide a moist environment	(48)
	Gauze dressings	Antibacterial agent	Bacterial protection is lost when the outer surface of the dressing becomes moistened by wound exudates; Gauze dressings tend to become more adherent to wounds as fluid production diminishes and are painful to remove causing patient discomfort	(49)
Modern dressings	Hydrocolloid dressings	Used for light to moderately exuding wounds and dry to moist wounds	Fibers are deposited in the wound and often have to be removed during dressing change	(50)
	Film dressings (nylon)	They are flexible; Provides moist healing environment; Promote autolytic debridement; Protect the wound from bacterial invasion and mechanical trauma	Limited ability to absorb sufficient quantities of wound exudates which results in the accumulation of excess exudates beneath the dressing; Tend to wrinkle on removal from their packs; Too thin to be packed into deep or cavity wounds and only suitable for relatively shallow wounds	(51, 52)
	Foam dressings	Maintains moist environment around the wound; Provides thermal insulation and highly absorbent	Not suitable for dry epithelializing wounds or dry scars	(53)
	Biological dressings	Forms part of the natural tissue matrix and are biodegradable	More research needs to be carried out using standard wound dressing for comparison	(54, 55)
	Tissue engineered skin substitutes	Used for delivery of bioactive materials such as growth factors and genetic materials to a wound	It cannot replace lost skin; High costs, risk of infection carry over and antigenicity	(56, 57)
Medicated dressings	Growth factors	Promotes wound healing through stimulation of angiogenesis and cellular proliferation	Choice of suitable and appropriate dressing is challenging for effective release and action at the wound site	(58–60)
	Antimicrobials	Treat infections	High antibiotic doses result in toxic reactions such as the cumulative cell and organ toxicity	(61, 62)
	Supplements (vitamins and mineral supplements)	Facilitate normal physiological wound healing	Limitations based on delivery of supplements to wounds from dressings is sparsely reported in the literature	(63–65)
Controlled drug delivery dressings	Polymeric drug delivery dressings	Serve as vehicles for the release and delivery of drugs to wound sites	Erosion of the polymer matrix following water diffusion and swelling in other dosage forms	(66, 67)

## Role of Complementary and Alternative Medicines in Wound Treatment

Alternative medicine is any healing practice “that does not fall within the realm of conventional medicine.” The terms 'complementary medicine' or 'alternative medicine' is used interchangeably with traditional medicine in some countries (68). It follows the traditional knowledge of ancient people or communities that they have been practicing for many years. As there was no modern or scientifically approved medicine in pre-historic times, CAMs were the only available option that was used universally to treat many diseases including wounds.

It is estimated that 30–50% of the general adult population of industrialized nations use one or the other forms of CAM, of which its use is more common among females, young adults/middle-aged individuals, members of higher socioeconomic classes, and highly educated people (69, 70). Internationally, studies on cancer patients documented a prevalence rate of 7–83% for the use of CAMs (70, 71).

Diseases have always been a great topic of concern in human society and human beings have been continuously involved in developing all sorts of possible treatment options to address the ailment. Recent publications reveal that doctors do not consistently record CAM at patient charts (72). A study that involved a patient with prostate cancer found a high usage of CAMs which had not been identified by conventional medication history taking (73). This suggests that the prevalence of CAM use among patients admitted to the hospital is high, but documentation of usage is low. This necessitates the need to record the history of CAM use by health professionals.

CAMs such as Natural Products including plant-derived extracts (phytochemicals) and Naturally Derived Substances have gained major research interest. Natural products and Naturally Derived Substances have long been used in wound healing because they possess anti-inflammatory, antioxidant, angiogenic, and cell synthesis-modulating properties. Alternative medical systems such as naturopathy and Ayurveda utilize herbal medications as an important part of therapy. Herbal medicines include any part of the plant, herbs, herbal materials or combinations, herbal extracts, and purified herbal products that contain an active ingredient called phytochemical. In India, Ayurvedic medicine has used wide variety of herbs including turmeric possibly as early as 1900 BC (74). There are a lot of studies and evidence that are supportive of the use of natural products and Naturally Derived Substances in wound care. Due to this reason, CAMs are currently gaining major attention by most of the researchers because of its promising results on patients and in wound care.

Phytochemicals and naturally derived substances have gained an advantage due to their composition of a plethora of chemicals that may enhance wound healing in many different ways. Using plants with medicinal properties to treat wounds have been found useful in fighting against infection and accelerate wound healing (75). A recent study conducted by Nigussie et al., described *Lawsonia inermis* and *Azadirachta indica* were the most studied plant species for wound healing, and the most common *in vivo* techniques used for the anti-inflammatory and the wound healing assays were carrageenan-induced paw edema, and excision and incision wound models, respectively (76). CAM is not only used for treating non-oncologic cutaneous problems but is also used in the prevention and treatment of malignant diseases (77). Detailed role of phytochemicals /naturally derived substances in wound healing studies with their uses, applications, mechanism of action, and outcome demonstrated in a particular study is discussed in Table 3. At the same time, the presence of numerous chemicals in an extract can limit the ability of the researcher to conclude the specific action of the individual chemical and their mechanism of action (101). However, their complex molecular structure and composition may increase the risk of irritant or allergic side effects. Thus, both patient and practitioner need to be cautioned of potential side effects.

**Table 3:** Comprehensive details on role of phytocompounds /naturally derived substances in wound healing studies.

**Name of Phytocompound/ Naturally derived substance**	**Type of formulation**	**Uses and applications in wound healing**	**Possible mechanism of action**	**Type of study or wound model used**	**Outcome**	**Reference**
Vitamin A	Topical and systemic	Anti-inflammatory property; Necessary for growth, differentiation, and maintenance of epithelial tissues	Influences morphogenesis, epithelial cell proliferation and differentiation in time and dose dependent manner	Preclinical and clinical	Prevents and treats infectious as well as inflammatory skin diseases	(78)
Vitamin E	Oral and topical	Used for resurfacing of the skin	Modulate cellular signaling, gene expression	Diabetic rats	Increased wound contraction	(79)
Vitamin C	Plant extract	Modulators of angiogenesis & collagen production; critically important for tensile strength of a wound	Act through induction of protein-kinase-C-dependent pathway that activates protein-1 DNA binding activity; hydroxylation of lysine and proline during the synthesis of collagen	*In vitro*	Stimulated growth of keratinocytes	(80, 81)
Alkaloids	Topical	Anti-inflammatory effects	Stimulate the growth of colonies from fibroblast precursors	*In vitro* and *in vivo*	Promotes early phases of wound healing (≤7 days) in a dose-dependent manner	(82)
Silymarin (polyphenol)	Topical ointments	Antioxidant properties	Help to prevent oxidative damage, increase epithelialization of wounds	Streptozotocin-induced experimental diabetic rats	Reduced inflammation in the wound that promoted the healing process	(83, 84)
Flavonoids	Pure Phytocompound/extract	Antioxidant, anti-allergic, anti-carcinogenic, anti-viral and anti-inflammatory agents	Involve hydrogen bonding and hydrophobic interactions	*In vitro*	Collagen fibers treated with catechin are stable	(85)
Tannins (phenolic compound)	Topical ointments	Act as astringents	Astringent property is responsible for wound contraction and increased rate of epithelialization at the granulation formation and scar remolding phases	*In vitro* and preclinical	Significant effect in wound closure and wound healing rate	(86, 87)
Terpenoids	Topical	Modulators of cytokines and growth factors	Increase in cell migration; increased collagen synthesis and tensile strength of wound tissues	Diabetic animals and clinical	Enhanced rate of wound healing	(88, 89)
β-sitosterol	Extract	Plant-derived angiogenic factor	Stimulates neovascularization and motility of human umbilical vein endothelial cells	*In vitro* (chick embryo)	Showed potent angiogenic activity	(90)
Kaempferol and quercetin (flavonoid)	Extract	Promising compounds for scar reduction; Regulators of extracellular matrix	Inhibition of fibroblast activities	*In vitro* and *in vivo*	Reduced scar formation	(91)
Aloe vera	Topical gel	Treat various ailments of the skin because of its antimicrobial and anti-inflammatory properties	Stimulate the release of several growth factors	Clinical studies	Aloe vera appeared to be helpful in acute wounds, but more controlled clinical studies will be needed to better assess its role in chronic wounds	(92)
Cocoa	Topical	Treat various ailments of the skin	Improves re-epithelialization	Porcine model	Improved wound healing, but limited studies have claimed the above results	(93)
Honey	Topical	Anti-inflammatory, antioxidant, antimicrobial, and osmotic properties	Wound healing effects are due to its antibacterial action, high acidity, osmotic effect, anti-oxidant, and hydrogen peroxide content	Clinical studies	Manuka honey showed higher activity against MRSA and vancomycin-resistant Enterococcus; MEDIHONEY a Manuka honey-derived product received FDA approval for the treatment of wounds; Honey was not found to benefit chronic venous leg ulcers; Lack of statistical evidence to prove the use of honey in superficial and partial thickness burn wounds	(94–98)
Neem	Extract	Irrigating agent	Increase in wound closure	Human diabetic foot ulcer patients	Enhanced wound healing	(99)
Banana leaf	Leaves	Dressings for partial thickness burn wounds and donor sites.	Enhances re-epithelialization	Human	Enhanced wound healing in unrandomized, controlled studies and no allergic or irritant reactions were observed	(100)

A study conducted by Raja (101) has shown that plant and naturally derived substances have a synergistic effect for wound healing. However, even in this study, side effects such as irritation and allergic hypersensitivities were noted (101). A recent study showed that neem leaf extract can be used as an alternate to normal saline and neem leaves extract irrigation for foot ulcers is considered to be very safe as it did not cause any complication systematically (99). Further, phytochemicals and naturally derived substances may possess greater risk of contamination with infectious agents. Thus, proper sterilization and timely microbial testing are necessary before their use. In this direction, collaborative research combining allopathic medicine with Ayurveda and naturopathy will provide a better understanding of how to mitigate the above-said limitations of natural products. This can further be integrated using a combined approach in wound treatment. In one of our studies, we have shown that phytochemical (constituting 44% w/w) especially tannins and other polyphenols in rind of Pomegranate (*Punica granatum*) can significantly heal the excision wound in experimental animals. Content of hydroxyproline, microscopic observation and physical appearance of wound are used as key markers to assess the benefit of pomegranate peel extract which was applied on would in the form of water-soluble gel (102). Our earlier reports also describe the use of various plant species and nano phytocompounds for various wound healing activities (103). One of the recent systematic review and meta-analysis on use of CAM in common skin diseases such as acne, atopic dermatitis and psoriasis reported that, there is insufficient evidence to support the efficacy and the recommendation of CAM for common skin diseases (104). However, as discussed earlier there are many ongoing studies that are reported in literature on the use of CAM in wound healing and their applications. This suggests that CAM has a potential future in chronic wound management.

## Conclusions and Future Directions

**Figure 3:**
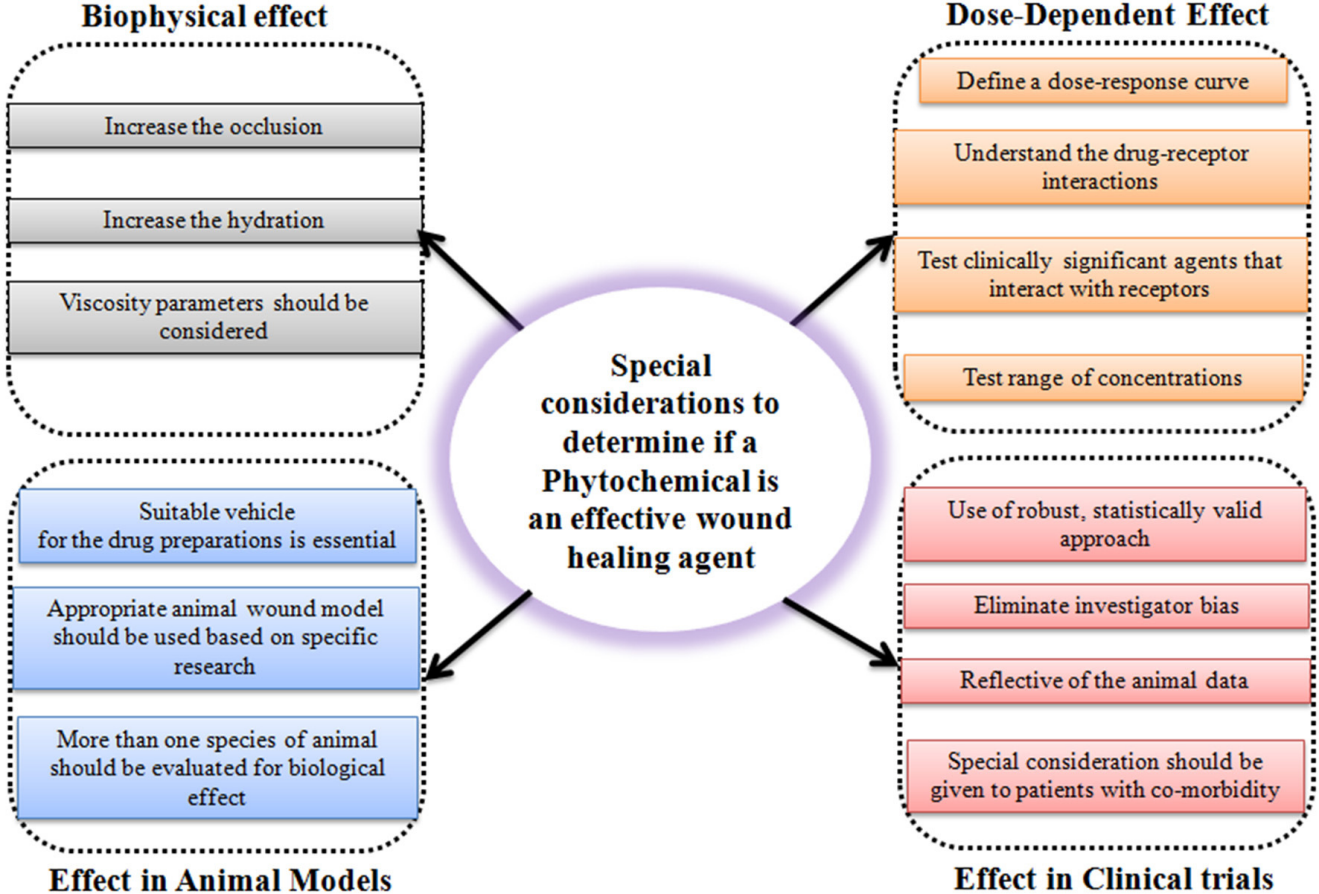
Schematic representation showing considerations to determine the efficiency of a phytochemical as a potential wound healing agent.

The use of phytochemicals and naturally derived substances is an exciting and clever innovation for chronic wound healing. CAM is a promising approach to improvising clinical and medical challenges faced by non-healing chronic wounds. However, it is the responsibility of a researcher to practically consider special factors to determine if the phytochemicals are an effective wound healing agent or not (Figure 3). Unfortunately, one of the major barriers to effective treatment of the wound seems to be the lack of deeper knowledge shown by many clinicians and general practitioners for this subject. CAMs are subject to the risk of contamination, side effects, and non-specificity in treatment due to the complex structure of phytochemicals and naturally derived substances in the extract. But these limitations do not deter the fact that CAMs are promising in challenging non-healing wounds. It is well-known that natural molecules possess anti-inflammatory, antioxidant, angiogenic, and cell synthesis-modulating components that are crucial biological functions necessary for wound healing. In addition to this, the use of natural products and naturally derived substances are considered safe compared to synthetic molecules and can be much cheaper than conventional therapies. Due to the escalating cost of health care especially in chronic wound management, the use of CAMs to treat these wounds would be economical. As they have multiple advantages over synthetic molecules, there is a spur in the use of natural molecules in wound healing research. However, more randomized clinical trials need to be carried out to provide concrete evidence to support the utilization of CAMs in management of chronic wounds. And, more research is needed to understand their mechanisms of action.

## Author Contributions

KC conceptualized the idea and wrote initial outline of the manuscript. PM wrote the entire manuscript. MC, AR, and PW critically reviewed the manuscript and provided suggestions. All authors have read, revised, and approved publication of the manuscript.

## Conflict of Interest

The authors declare that the research was conducted in the absence of any commercial or financial relationships that could be construed as a potential conflict of interest.

## Publisher's Note

All claims expressed in this article are solely those of the authors and do not necessarily represent those of their affiliated organizations, or those of the publisher, the editors and the reviewers. Any product that may be evaluated in this article, or claim that may be made by its manufacturer, is not guaranteed or endorsed by the publisher.
